# Breast and prostate cancer patients differ significantly in their serum Thymidine kinase 1 (TK1) specific activities compared with those hematological malignancies and blood donors: implications of using serum TK1 as a biomarker

**DOI:** 10.1186/s12885-015-1073-8

**Published:** 2015-02-18

**Authors:** Kiran Kumar Jagarlamudi, Lars Olof Hansson, Staffan Eriksson

**Affiliations:** 1 Department of Anatomy, Physiology, and Biochemistry, Veterinary Medicine and Animal Science center, Swedish University of Agricultural Sciences, P.O. Box 7011,, S-75007 Uppsala, Sweden; 2 Department of Laboratory Medicine, Karolinska Institute, Stockholm, Sweden

**Keywords:** Serum thymidine kinase 1, STK1 protein assays, Size exclusion chromatography, Anti-human TK1 antibodies, TK1 specific activity

## Abstract

**Background:**

Thymidine kinase 1 (TK1) is a cellular enzyme involved in DNA precursor synthesis, and its activity has been used as a proliferation marker for monitoring malignant diseases. Here, for the first time, we evaluated both TK1 activity and protein levels in sera from patients with different malignancies.

**Methods:**

Serum samples from patients with myelodysplastic syndrome (MDS, n = 22), breast cancer (n = 42), prostate cancer (n = 47) and blood donors (n = 30) were analyzed for TK1 protein and activity levels, using a serum TK1 (STK1) protein assay based on antibodies and an activity assay that measured [^3^H]-deoxythymidine (dThd) phosphorylation. The molecular forms of TK1 in sera from some of these patients were analyzed using size-exclusion chromatography.

**Results:**

Mean STK1 activities in sera from MDS, breast and prostate cancer were 11 ± 17.5, 6.7 ± 19 and 1.8 ± 1.4 pmol/min/mL, differing significantly from blood donors (mean ± standard deviation (SD) = 1.1 ± 0.9 pmol/min/mL). Serum TK1 protein (25 kDa polypeptide) levels were also significantly higher in MDS, breast, prostate cancer compared to blood donors (mean ± SD = 19 ± 9, 22 ± 11, 20 ± 12, and 5 ± 3.5 ng/mL, respectively). The STK1 specific activities of sera from patients with MDS and blood donors were significantly higher when compared with activities in sera from breast and prostate cancer patients. Size-exclusion analysis of sera from breast and prostate cancer showed that the detected active TK1 was primarily a high molecular weight complex, similar to the forms found in sera from MDS patients and blood donors. However, Western blotting demonstrated high TK1 25 kDa protein levels in fractions lacking TK1 activity in sera from cases with breast and prostate cancer.

**Conclusions:**

These results demonstrate that there are differences in the specific activities and the subunit compositions of STK1 in hematological malignancies compared with breast and prostate cancer. This fact has several important implications for the use of STK1 as a tumor biomarker. One is that STK1 protein assays may differentiate early-stage tumor development in breast and prostate cancer more effectively than STK1 activity assays.

**Electronic supplementary material:**

The online version of this article (doi:10.1186/s12885-015-1073-8) contains supplementary material, which is available to authorized users.

## Background

Tumors are primarily characterized by uncontrolled cell proliferation, and the proliferative activity of cancer cells correlates with the aggressiveness of the disease. Prognostic markers that can measure tumor-cell proliferation are clinically valuable because they may improve the early detection and treatment monitoring of tumor diseases [1]. Thymidine kinase 1 (TK1) is one of these proliferation biomarkers and is involved in the salvage pathway of DNA precursor synthesis. TK1 catalyzes the conversion of thymidine to deoxythymidine monophosphate (dTMP), which is further phosphorylated to di (dTDP) and triphosphates (dTTP) prior to being incorporated into DNA [2]. TK1 expression is S-phase dependent, and high levels of TK1 have been noted in proliferating and malignant cells [3,4]. TK1 activity increases in late G1 and peaks in the S phase and then decreases during the M phase due to the action of a specific degradation pathway [5]. STK1 (serum TK1) activity may be measured using different assays, e.g., TK-REA [6], TK Liaison [7], the Divitum assay [8] and the [^3^H]-deoxythymidine (dThd) phosphorylation assay [9]. Recent study showed that [^3^H]-dThd phosphorylation correlates well with the TK-Liasion assay (r = 0.96) and TK-REA (r = 0.92) [9]. It is well established that STK1 activity may be used as a prognostic marker in cases of leukemia and lymphomas [10-13] and to some extent in cases of breast cancer [14-16].

The development of anti-human TK1 antibodies [17] has extended the application of serum TK1 protein determination for many different tumor diseases, and several clinical studies have demonstrated increased serum TK1 protein levels in solid-tumor diseases [18-23]. In general, TK1 protein assays showed higher sensitivity than STK1 activity measurements in cases with solid-tumor diseases, both in humans [24] and dogs [25].

The concentration of STK1 correlates with diagnosis and treatment outcome in breast [26-28], lung and gastric carcinomas [29,30]. However, STK1 activities do not show this pattern. A recent study demonstrated that TK1-ELISA is more sensitive for early-stage detection of lung cancer compared with STK1 activity assays [31]. In this study, we measured both serum TK1 activity and TK1-25-kDa protein levels in MDS, breast and prostate cancer patients. The ability of the STK1 activity assays and STK1 protein assays to discriminate malignant samples from blood donors was tested using ROC analysis. The specific activities of serum TK1 protein in the sera from these patients and blood donors were determined. The molecular forms of TK1 in some of these sera samples were also investigated using size-exclusion chromatography, and important differences were revealed.

## Methods

### Serum samples

Serum samples from MDS (n = 22), breast (n = 42) and prostate cancer (n = 47) patients were purchased from Biotheme Research Solutions Inc., Florida, USA (Samples were collected as de-identified diagnostic remainders excempt from Title 46, Title 21 and HIPAA IRB/ Consent requirements. Serum samples were collected under an IRB approved protocol or collected as consented donor samples from FDA licensed/registered facility following GMPs. They followed necessary procedures for obtaining the informed consent of donors). Information on the clinical staging (TNM), histological classification for breast cancer patients and the Gleason Score (GS) in cases with prostate cancer were received from Biotheme and are shown in the supplementary tables. Serum samples from blood donors (n = 30) were purchased from ProMedDx, Norton, MA, USA, and they were free from any viral infections or illnesses. The mean age was 27 years (range 17-59 years) for the blood donors, MDS patients showed a mean age of 76 years (range 61-89), whereas the mean age was 58 years (range 43-86 years) for patients with breast cancer. In cases with prostate cancer, the mean age was 70 years (range 33-91 years). Samples were stored at -20°C until analysis. The study comprised 141 samples including sera from blood donors (n = 30) and patients with MDS (n = 22), breast cancer (n = 42) or prostate cancer (n = 47).

### TK1 antibodies, chicken polyclonal and mouse monoclonal XPA 210 antibodies

Both monoclonal and polyclonal antibodies used in this study were raised against the C- terminal region of TK1. A 31-amino acid peptide (GQPAG PDNKE NCPVP GKPGE AVAAR KLFAPQ, 194-225) was used the antigen for the production of chicken immunoglobulin (IgY) antibodies, and they were purified by affinity chromatography as described previously [17]. Mouse monoclonal antibody (XPA 210) against the same peptide was prepared as previously described [4]. These antibodies were supplied by Arocell AB (Uppsala, Sweden).

### Serum thymidine kinase activity assay

STK1 activities in the serum samples were determined using an optimized [^3^H]-dThd phosphorylation assay as described previously [9]. In brief, 10 μL of serum was incubated with a reaction buffer containing 20 mM Tris/HCl, pH 7.6, 2 mM dithiothreitol (DTT), 5 mM sodium fluoride (NaF), 5 mM MgCl_2_, 5 mM adenosine triphosphate (ATP) and 5 μM [-^3^H]-dThd (20 Ci/ml, PerkinElmer, Boston, MA, USA). The reaction mixture was incubated at 37°C for 1 h. The radioactivity in the reaction products was determined as described previously [9]. Thymidine kinase 1 activities were expressed as pmol dTMP formed per min per mL of serum.

### Immunoaffinity Sepharose preparations

**Figure 1:**
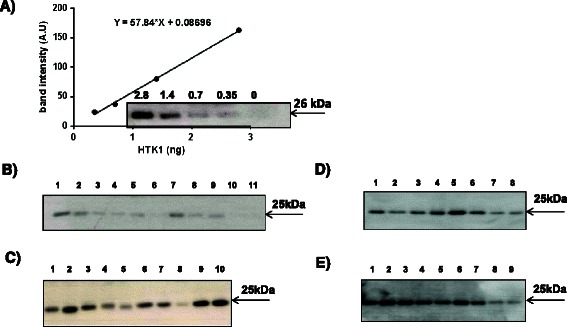
**Immunoaffinity analysis of human recombinant TK1 and serum TK1. (A)** Results from the immunoaffinity/Western blotting analysis of purified recombinant human TK1 and the 26 kDa band relative intensity (arbitrary units, A.U.) used as a standard. **(B)** Examples of results of the immunoaffinity/Western blotting analysis of sera from blood donors, **(C)** MDS patients, **(D)** breast cancer, **(E)** or prostate cancer patients.

The XPA 210 antibody was covalently coupled to cyanogen bromide (CNBr)-activated Sepharose 4 fast flow (GE HealthCare, Uppsala, Sweden) as described previously [25] and as shown in the supplementary information (Figure 1). The antibody-coupled Sepharose was stored in Tris-buffered saline (TBS) containing 0.01% NaN_3_ 1:1 at 4°C.

### Isolation of thymidine kinase 1 (TK1) using anti-TK1 antibody-coupled Sepharose

Recombinant human TK1 (0.3-2.8 ng), 50 μL of serum samples and 300 μL of FPLC fractions from serum samples were diluted with 180μL of TBS, pH 7.6, and then incubated with anti-human TK1 antibody-coupled Sepharose (70 μL of a 1:1 V/V mixture of antibody-Sepharose and TBS). The samples were then agitated at 4°C for 4 h followed by centrifugation for 1 min at 13,000 revolutions per min (RPM). The antibody-coupled Sepharose was washed twice with TBS, once with TBS-T and one more time with TBS. Then, 30 μL of sample buffer (containing Tris-HCl, pH 6.8, 0.5 M; glycerol, 20% (w/v) SDS, 10%; bromophenol blue 0.1%; and DTT,10 mM) was added to the Sepharose and the solution incubated at room temperature for 20 min to elute bound serum proteins. These eluted complex proteins were heated at 95°C for 5 min and analyzed using 12% SDS-PAGE and transferred to PVDF membranes (GE-Healthcare) using a semi-dry transfer method. After blocking with 5% BSA in TBS-Tween for 1 hr, the bound proteins were detected using a chicken anti-TK1 antibody followed by incubation with a HRP-conjugated anti-chicken IgY antibody. The peroxidase reaction was detected using ECL (GE Healthcare, Uppsala, Sweden). Films were exposed for 2 min to obtain detectable band intensities for serum and recombinant TK1 controls. Densitometry scanning of these films was performed using an Epson perfection V scanner. The TK1 polypeptide band corresponding to 25 kDa was quantified using image analysis software (Image Gauge G3.3, Science Lab, Fuji Photo Film Co., Ltd., Tokyo, Japan). A standard curve was created by measuring the intensities of different amounts of recombinant TK1.

### Size-exclusion chromatography

Size-exclusion chromatography was performed as described previously [32,33]. In brief, 200 μL of sera from MDS, breast, prostate cancer patients and blood donors was diluted to 1:1 in a buffer containing 0.01 M Hepes, 0.15 M NH_4_Cl and 0.02% NaN_3_ prior to injection into a Superose 12 column (1.0 × 30 cm, GE Health care, Sweden). The column was equilibrated with the same buffer and eluted at flow rate of 0.4 mL/min. Twenty four fractions (0.4 mL) were collected, and the TK1 protein content in the fractions determined and compared with standards of different molecular weights: α2-macroglobulin (720 kDa), β amylase (200 kDa), bovine serum albumin (66 kDa), ova albumin (45 kDa) and horse myosin (17 kDa).

### Statistical analysis

Distributions of STK1 activity and protein levels in healthy, MDS, breast and prostate cancer sera were evaluated for normality using the D’Agostino and Pearson omnibus normality test. Serum TK1 activities in different groups showed non-Gaussian distributions, whereas STK1 protein levels followed Gaussian distributions. Consequently, the Mann-Whitney U test was used for comparing STK1 activities between groups, and unpaired *t*-tests were used to compare differences in STK1 protein levels between groups. One way ANOVA followed by Turkey’s multiple comparison post-test to compare TK1 protein levels and Dunn’s Multiple Comparison post-test was used to compare TK1 activity across multiple groups. Spearman’s correlation coefficient (*rs*) was used to determine correlations between different parameters. All statistical analyses were performed using GraphPad Prism 5.0 (GraphPad Software, La Jolla, CA, USA). Receiver operating characteristic (ROC) curves were constructed to evaluate the performance of the [^3^H]-dThd phosphorylation assay and the immunoaffinity assay results in different groups. Statistical significance was achieved when *P* < 0.05.

## Results

### Serum sample STK1 activities and STK1 protein levels

Immunoaffinity Western blotting assays were established using different concentrations (0.3-2.8 ng) of human recombinant TK1 as standards (Figure 1A). Examples of determinations of the TK1-25-kDa protein bands in sera from blood donors and patients with MDS, breast and prostate cancer are shown in Figure 1B-E, respectively. Protein levels were determined using a recombinant TK1 standard curve. Blots of the remaining serum samples may be found in Additional file 1: Figures S2, S3 and S4. Some cross-reacting bands were detected in the high-molecular-weight region; however, the majority was due to cross reactivity originating from the secondary anti-chicken IgY antibodies. No cross-reacting bands were detected at 25 kDa, which was also demonstrated in competition experiments where the anti-TK1 antibody was mixed with the antigen peptide (at a 1:1 ratio) (data not shown). These results strongly suggest that the immunoaffinity assay provides an accurate measurement of STK1 protein levels in the samples.

The mean serum TK1 activities and TK1 protein levels for the blood donors and the different patient groups are shown in Table 1 and in Additional file 2: Tables S1-S4 and are described separately below.

**Table 1:** **Mean serum thymidine kinase 1 (STK1) protein, STK1 activity levels and correlation between STK1 protein and activity in different groups**

		STK1 protein (ng/mL)	STK1 activity (pmol/min/mL)		
		Mean ± SD (M)	Mean ± SD (M)		
Tumor type	N			rs*	*P*
Myeloid dysplastic	22	19 ± 9.0 (19)	11 ± 17.5 (3)	0.63	0.0015
Syndrome (MDS)					
Breast cancer	42	22 ± 11 (21)	6.7 ± 18.8 (1.8)	0.41	0.0077
Prostate cancer	47	20 ± 12 (22)	1.8 ± 1.4 (1.3)	0.45	0.0015
Healthy	30	5 ± 3.5 (4)	1.1 ± 0.6 (0.9)	0.49	0.0056

*Spearman’s correlation coefficient.

#### Blood donors

The mean STK1 activity values ranged from 0.5-3.2 pmol/min/mL (mean ± SD = 1.1 ± 0.6 and median = 1.0). Immunoaffinity/Western blotting analyses revealed faint TK1-25-kDa protein bands in the majority of the donor sera; however, approximately 30% were below the detection limit (Figure 1B). Thus, STK1 protein levels ranged from undetectable to 13 ng/mL (mean ± SD = 5 ± 3.5 and median = 4.0). A significant correlation was observed between STK1 activity and STK1 protein levels in the samples from blood donors (*rs =* 0.49, P = 0.005). In this experimental group, there were no significant changes in STK1 activity or STK1 protein levels with age.

#### MDS patients

**Figure 2:**
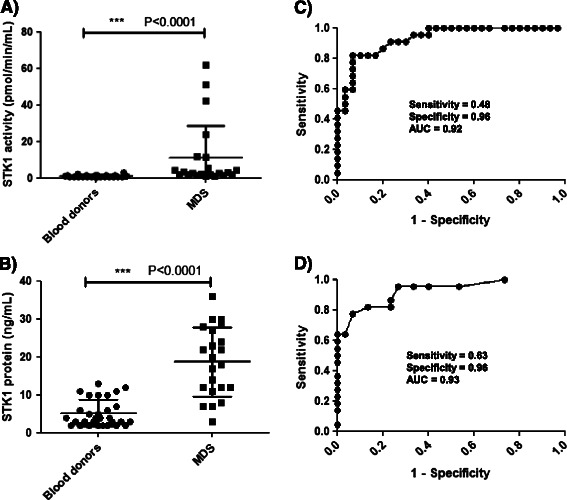
**TK1 activity and protein levels in sera from MDS patients and blood donors. (A)** STK1 activity in sera from blood donors (•) and MDS patients (■). **(B)** Concentrations of STK1 in sera from blood donors (•) and MDS patients (■). Error bars denote SDs. **(C)** The receiver operating characteristic (ROC) curve of STK1 activities for discrimination between blood donors and MDS patients. **(D)** ROC curve analysis of STK1 protein levels in blood donors and MDS patients.

The mean ± SD STK1 activity in MDS patient sera was 11 ± 17.5, with a range of 1-62 pmol/min/mL, which was significantly higher compared with blood-donor sera (P < 0.0001, Figure 2A). Western blotting analysis revealed TK1 protein levels with a range of 3-36 ng/mL (mean ± SD = 19 ± 9 and median = 19), which was significantly higher than in sera from blood donors (P < 0.0001, Figure 2B). No significant correlation was found between age and STK1 activity or STK1 protein levels. ROC curve analysis revealed that the TK1 activity assay showed an area under the curve (AUC) of 0.93, (P < 0.0001; 95% confidence interval (CI) 0.86-0.99), with a cutoff value of 3.1 pmol/min/mL, a sensitivity of 0.48 and specificity of 0.96 (Figure 2C). The TK1 protein assay showed an AUC of 0.93, (P < 0.0001; 95% CI 0.85-0.99), a cutoff value of 12.5 ng/mL, a sensitivity of 0.63 and a specificity of 0.96 (Figure 2D).

#### Breast cancer

**Figure 3:**
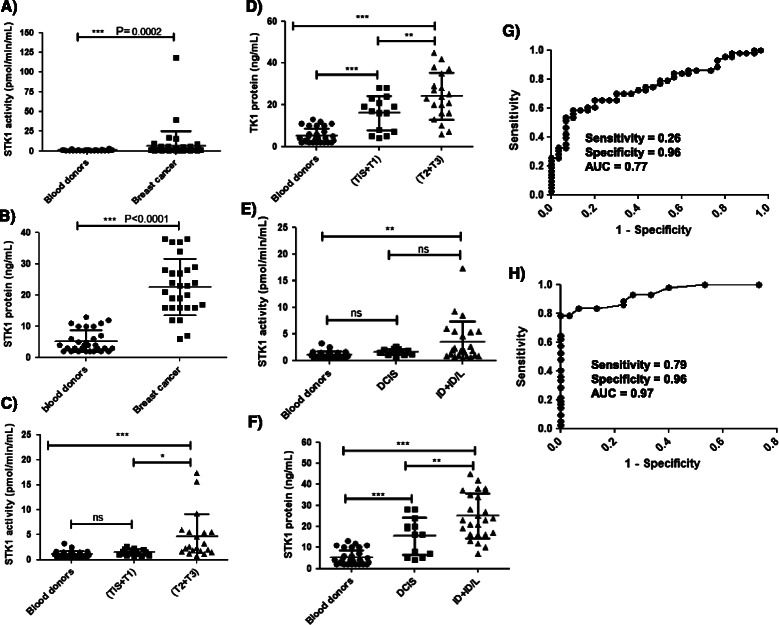
**TK1 activity and protein levels in breast cancer patients and blood donors. (A)** STK1 activity distributions in sera from blood donors (•) and patients with breast cancer (■). **(B)** STK1 protein levels in sera from blood donors (•) and patients with breast cancer (■). **(C)** Comparison of STK1 activities in sera from blood donors (•) and early-stage (TiS + T1; ■) and late-stage (T2 + T3; ▲) breast cancer patients. **(D)** Comparison of STK1 protein levels in sera from blood donors (•) and early-stage (TiS + T1; ■) and late stage (T2 + T3; ▲) breast cancer patients. **(E)** Comparison of STK1 activities in blood donors (•) and non-invasive (DCIS; ■) and invasive (ID + ID/L; ▲) breast cancer patients. **(F)** Comparison of STK1 protein levels in blood donors (•) and non-invasive (DCIS; ■) and invasive (ID + ID/L) breast cancer patients (▲). The error bars denote standard deviations (SDs). **(G)** Receiver operating characteristic (ROC) curve of STK1 activity in serum samples from breast cancer patients and from blood donors. **(H)** ROC curve analysis of STK1 protein levels in sera from blood donors and breast cancer patients.

STK1 activity levels in breast cancer sera were in the range of 0.5-118 pmol/min/mL (mean ± SD = 6.7 ± 19 and median = 1.8) and were significantly higher than levels in sera from blood donors (Figure 3A). Immunoaffinity/Western blotting analysis of sera from breast cancer patients showed high STK1 protein levels compared with blood donors, and they ranged up to 45 ng/mL (mean ± SD = 21 ± 10 and median = 21; Figure 3B). A significant negative correlation (*r*_*s*_ = -0.41, *P* = 0.0057) was observed between increasing age and STK1 activity; however, STK1 protein levels did not show this correlation in this patient group. Serum samples were classified into two groups based on tumor size (T), i.e., early stage (TiS + T1) and late stage (T2 + T3). STK1 activities were significantly higher in late-stage (T2 + T3) compared with blood donors (P < 0.0001) and early-stage (TiS + T1) patients (P = 0.0273). However, STK1 activity levels in early-stage patients (TiS + T1) were not significantly different compared with blood donors (P = 0.283) (Figure 3C), whereas STK1 protein levels from early (P = 0.0001) and late stages of tumor progression (P < 0.0001) were significantly different from blood donors (Figure 3D). These samples were further classified based on histological examinations [34] as ductal carcinoma in situ (DCIS), invasive ductal carcinoma (ID) and invasive ductal/ lobular carcinoma (ID/L). STK1 activity levels in invasive carcinoma (ID + ID/L) were significantly higher than in blood donors (P = 0.002) but this increase was not present in non-invasive (DCIS), which is considered an early stage of tumor development (P = 0.054) (Figure 3E). STK1 protein levels in invasive carcinomas (P < 0.0001) and DCIS were significantly higher than in blood donors (P = 0.0014) (Figure 3F). Furthermore, ROC curve analysis showed that the TK1 activity assay had an AUC of 0.77 (P < 0.0001, 95% CI = 0.66–0.87), with a cutoff value of 3.1 pmol/min/mL, a sensitivity of 0.26 and a specificity of 0.96 (Figure 3G). In the case of the TK1 protein assay, the AUC was 0.97 (P < 0.0001, 95% CI 0.90-0.99). At the optimal cutoff value of 12.5 ng/mL, the true positive rate was 79%, whereas the false-positive rate was 4% (Figure 3H). These results indicate that the STK1 protein assay may differentiate early-stage tumors more efficiently compared with the STK1 activity assay.

#### Prostate cancer

**Figure 4:**
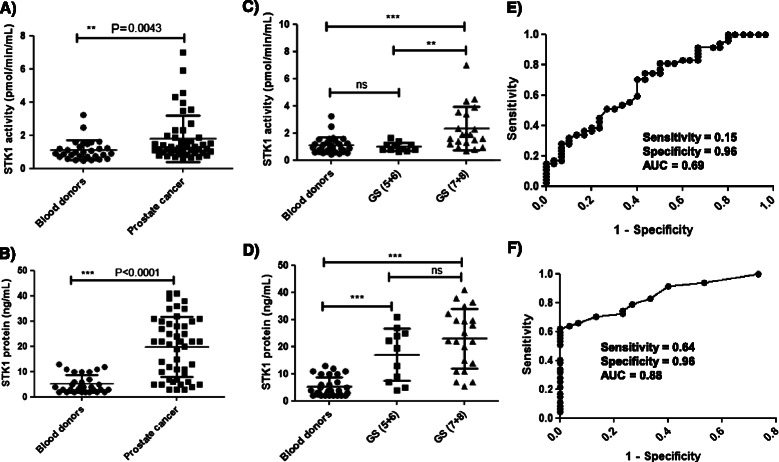
**TK1 activity and protein levels in sera from prostate cancer patients and blood donors. (A)** STK1 activity in sera from blood donors (•) and prostate cancer patients (■). **(B)** Concentrations of STK1 in sera from blood donors (•) and prostate cancer patients (■). **(C)** Comparison of STK1 activity in sera from blood donors (•) and well-differentiated (GS5 + 6; ■) and moderately/poorly differentiated (GS7 + 8; ▲) prostate cancer patients. **(D)** Comparison of STK1 protein levels in blood donors (•) and well-differentiated (GS5 + 6; ■) and moderately/poorly differentiated (GS7 + 8; ▲) prostate cancer patients. The error bars denote standard deviations (SDs). **(E)** Receiver operating characteristic (ROC) curve of STK1 activities in sera from prostate cancer patients compared with sera from blood donors. **(F)** A similar ROC curve analysis of STK1 protein assay results in sera from blood donors and prostate cancer patients.

In prostate cancer sera, STK1 activities ranged from 0.6-7 pmol/min/mL (mean ± SD = 1.8 ± 1.4 and median = 1.3), and they showed significantly higher STK1 activity than blood donors (Figure 4A). The mean STK1 protein levels in sera from prostate cancer patients (mean ± SD = 20 ± 12 ng/mL and median = 22) was significantly higher compared with levels in sera from blood donors (Figure 4B). Based on the modified Gleason score (GS) grades [35], prostate serum samples were divided into two categories: well-differentiated (GS5 + 6) and moderately/poorly differentiated (GS7 + 8) cancer. The STK1 activity assay discriminated moderately/poorly differentiated from well-differentiated cancer (P = 0.0094) and from blood donors (P = 0.0009) but no significant difference was observed between well-differentiated cancer and blood donors (P > 0.99) (Figure 4C). In contrast, the STK1 protein assay significantly discriminated well-differentiated cancer from blood donors (P = 0.0002) (Figure 4D). These results indicate that the STK1 protein assay, but not the STK1 activity assay, was capable of detecting well-differentiated cancer prior to its progression to poorly differentiated cancer. ROC curve analysis demonstrated that the STK1 activity assay had a sensitivity of 0.15 and a specificity of 0.96 with an AUC of 0.69 (*P* = 0.0042, 95% confidence interval (CI) 0.57–0.81; Figure 4E) at the optimal cutoff value of 2.9 pmol/min/mL. The STK1 protein assay showed an AUC of 0.88 with a sensitivity of 0.64 and a specificity 0.96 (P < 0.0001, 95% confidence interval (CI) 0.81–0.95) at a cut-off value of 12.5 ng/mL (Figure 4F).

**Figure 5:**
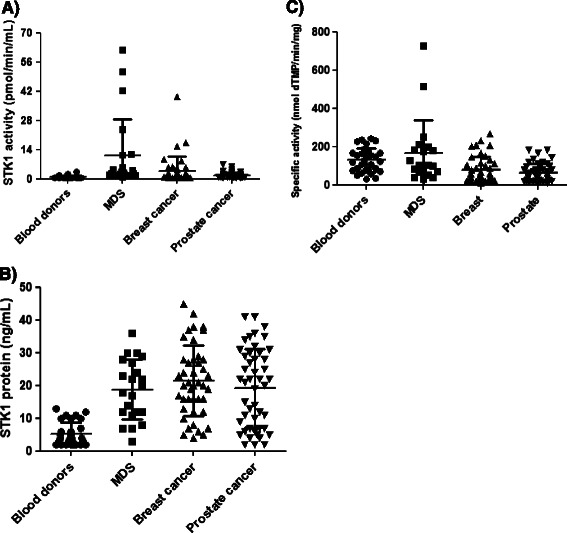
**Serum TK1 activity, STK1 protein levels and STK1 specific activities in different groups. (A)** STK1 activities in sera from blood donors (•), MDS (■), breast (▲) and prostate cancer patients (▼). **(B)** STK1 protein levels in sera from blood donors (•), MDS (■), breast (▲) and prostate cancer patients (▼). **(C)** Specific activities of TK1 in sera from blood donors (•), MDS (■), breast (▲) and prostate cancer patients (▼).

Serum TK1 activity values (Figure 5A) and STK1 protein levels (Figure 5B) in healthy, MDS, breast and prostate cancer were compared, and no significant differences were found in mean STK1 protein levels between MDS, breast and prostate cancer. However, significant correlations were found between STK1 activity and STK1 protein levels in MDS, breast and prostate cancer sera (*r*_*s*_ = 0.63, *P* = 0.0015, *rs* = 0.41, *P* = 0.0077 and *rs* = 0.45, *P =* 0.0015; Table 1).

### The specific activity of TK1 in sera from different groups

In this study, the specific activity of STK1, i.e., the nmol dTMP formed per min per mg of STK of 25 kDa protein, was determined using a significant number of clinical samples. The specific activities are based on direct [^3^H]-dThd phosphorylation activity measurements and on determinations of STK1 protein levels using immunoaffinity/Western blotting analyses. The results are shown in Table 2 and may be summarized as follows: the specific activity of STK1 (25 kDa) protein (mean ± SD) in sera from blood donors was 133 ± 67 nmol/min/mg, in sera from MDS patients, it was 137 ± 112, in breast cancer sera, it was 61 ± 53 and in prostate cancer sera, it was 52 ± 35 nmol/min/mg. Specific activities are compared in Figure 5C, and the highest STK1 specific activities were noted in three sera from MDS patients (sample no: 10, 15 and 20, Additional file 2: Table S2) and in two sera from breast-cancer patients (sample no: 9 and 37, Additional file 2: Table S3). Because these samples showed very high values, they were regarded as outliers and were excluded from the mean of the groups. The mean specific activity of TK1 in sera from blood donors and MDS patients was significantly higher (approx. 2-fold) compared with activities in sera from breast and prostate cancer patients (Table 2).

**Table 2:** **Mean serum thymidine kinase 1 (STK1) specific activity values in different groups and comparison with blood donors**

		STK1 specific activity (nmol/min/mg)			
Tumor type	N	Mean ± SD	Range (nmol/min/mg)	Comparison with blood donors	P value
MDS	22	136 ± 112	30 -515	no signifcant difference	0.635
Breast cancer	42	79 ± 69	12 -268	Yes (***)	0.0005
Prostate cancer	47	62 ± 47	11 - 183	Yes (***)	<0.0001
Blood donors	30	130 ± 62	29 - 243		

*indicates the significant difference levels (* = low, ** = medium, *** = high).

### Molecular forms of serum TK1 in patients with different malignancies

**Figure 6:**
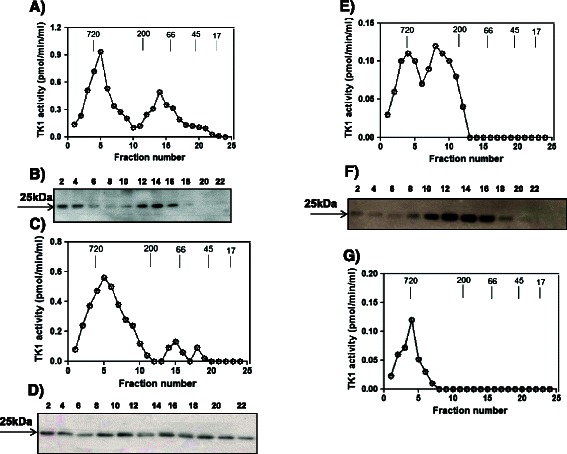
**Molecular forms of serum TK1 analyzed using size-exclusion chromatography. (A)** Thymidine kinase 1 activity in serum fractions from an MDS patient. **(B)** Western blotting analysis of MDS serum fractions using the immunoaffinity method. **(C)** Thymidine kinase 1 activity in serum fractions from a patient with breast cancer. **(D)** Western blotting analysis of serum fractions from a patient with breast cancer using the immunoaffinity method. **(E)** Thymidine kinase 1 activity in serum fractions from a patient with prostate cancer. **(F)** Western blotting analysis of serum fractions from a prostate cancer patient using the immunoaffinity method. **(G)** Thymidine kinase 1 activity in fractions from the serum of a blood donor. The numbers denote FPLC fractions. Arrows denote the elution position with respect to molecular weight markers.

A serum sample from MDS patient no. 20, (Additional file 2: Table S2), a breast cancer serum sample (sample no. 37, Additional file 2: Table S3), a prostate cancer sample (sample no. 28, Additional file 2: Table S4) and a blood-donor sample (no. 29, Additional file 2: Table S1) were analyzed using size-exclusion chromatography. In the MDS sample, TK1 activity appeared in two peaks, one in the MW range of 400-720 kDa and the other in the MW range 50-200 kDa (Figure 6A). A TK1 polypeptide of 25 kDa was also observed in two peaks, similar to TK1 activity, however, in this experiment, the TK1 protein levels did not correlate exactly with activity in the fractions (Figure 6B, Additional file 1: Figure S5A). In serum from the breast cancer patient, 90% of total TK1 activity eluted in fractions corresponding to MWs of 300-720 kDa (Figure 6C) with two minor peaks observed in the MW range 200-50 kDa. However, the immunoaffinity/Western blotting analysis revealed a TK1 polypeptide of 25 kDa in almost all fractions (Figure 6D) with no apparent correlation between band intensity and TK activity (Additional file 1: Figure S5B). In serum from a prostate cancer patient, very low TK1 activity levels were noted in fractions corresponding to the MW range 200-720 kDa (Figure 6E). However, the TK1-25-kDa protein showed a peak in fractions corresponding to MWs of 200-40 kDa. There was also a low level of 25-kDa protein in the high-MW fractions where a TK1 activity peak was observed (Figure 6F). In the sample from the blood donor, a TK1 activity peak eluted in the high-MW region (Figure 6G), and no protein bands were detected (data not shown).

These results demonstrate that serum TK1 occurs in multiple oligomeric forms and that TK1 activity was associated with the very high MW complexes, whereas TK1 protein was noted in many different molecular forms (from 40-700 kDa), with no apparent correlation to TK1 activity peaks. These experiments were repeated using serum samples from one additional MDS (sample no. 15), breast cancer (sample no. 20) and prostate cancer (sample no. 27) patient and a blood donor (sample no. 17), and in general, very similar results were observed, as shown in Additional file 1: Figure S4. Therefore, we consider the different distributions in TK1 activity and TK1 protein levels described above for MDS, breast and prostate patients as being representative for the respective patient groups.

## Discussion

Serum TK1 activity measurement has been used as tumor proliferation marker for the diagnosis and prognosis of various hematological malignancies [11,12] and in some solid-tumor diseases [8,15]. Development of TK1 antibody-based methods demonstrated that the concentration of STK1 protein may be used for the prognosis and monitoring of tumor therapy in several types of solid tumor diseases [19,21,23,36]. In this study, we attempted to clarify the reasons underlying the apparently different information in different forms of malignant diseases that measurement of STK1 activities and TK1 protein levels provide. We took advantage of the fact that we have sensitive assays for determining STK1 activities and STK1-25-kDa protein levels. Thus far, no studies have been conducted where the specific activities of STK1 or the molecular forms of TK1 in different types of tumor diseases have been analyzed in detail.

Patients with myelodysplastic syndrome (MDS) are classified into many subtypes but all have a dysfunctional blood-forming capacity, more specifically in regeneration of myeloid linages, with a large fraction of patients progressing to acute myeloid leukemia. In the patients tested here, increased STK1 activities and 25 kDa protein, above the cut-off (12.5 ng/mL) of the blood donor control group, was observed in 15 out of 22 cases. This number is surprisingly high, particularly because the control group was not age-matched and was of a mean age of 27 compared with the mean age of 76 y in the MDS group. Earlier studies have shown a decrease in mean STK1 activity and protein levels with increasing age [19]. The specific activity of STK1 in the MDS patients varied from 30 to 858 nmoles per min per mg TK 25 kDa protein; however, the mean value was similar to that of the blood donor group. The mechanism for the variation and similarities between blood donors and MDS patients in specific activities and molecular form will be discussed below. Here, we have studied 111 clinical samples from patients who were diagnosed with MDS, breast or prostate cancer. Particularly in breast cancer patients, the availability of clinical data on staging and histological grad enabled comparisons between the two assays. Similarly, Gleason scores (GS) of the prostate cancer patients made a preliminary clinical assessment of the STK1 protein assay possible. Furthermore, these results indicate that the STK1 protein assay may distinguish early stages of cancer diseases more efficiently compared with the TK activity assay. The principles and basic performance of the two STK1 assays used here have been described previously [9,25].

Consistent with earlier studies using TK-Liaison and Divitum TK activity assays [37], we observed that STK1 activity values in sera from breast and prostate cancer were higher than the healthy cut-off in approximately 25% of samples. This finding was also evident from our ROC curve analysis, which showed a sensitivity of 26% with 96% specificity in the case of breast cancer. However, with respect to STK1 protein levels, immunoaffinity/Western blotting assays distinguished breast cancer from blood donors with sensitivity close to 79%at 96% specificity. In the case of prostate cancer patients, the results were similar, with the STK1 protein assay improving sensitivity 3-fold compared with the activity assay. Previous studies demonstrated that in sera from patients with leukemia, gastric and breast cancer and healthy controls, there was a significant correlation between TK1 protein concentration and TK1 activity [24]. Similar results were found in this study, and a possible explanation for these correlations may be that the protein assay measures both active and inactive serum TK1, whereas the activity assay measures only the active high-MW TK1 protein complexes. The results presented here are consistent with our recently published results on STK1 determination in dogs with malignant diseases. We observed no significant difference in STK1 activities in sera from dogs with solid tumors compared with healthy dogs. However, there was a significant difference in STK1 protein levels between these two groups [25]. Although the clinical application of TK1 as a biomarker has increased in recent years, very few studies have been conducted to determine the molecular forms of TK1 in different forms of malignancies [32,33]. Here, we used size-exclusion chromatography to investigate this question. The most significant and unexpected finding was that serum TK1 activity and TK1 protein did not clearly co-elute in any of the sera analyzed from patients with malignancies. The situation in blood donors was not resolved due to the fact that TK1 protein was not detected in the fractions containing enzyme activity. Sera from MDS patients showed two peaks of TK1 activity and the TK1 protein; however, the patterns were not identical. The discrepancies between TK1 activity and protein-elution profiles were even more apparent in sera from breast and prostate cancer patients, where STK1 activity was primarily observed as an active high-MW complex, whereas the TK1 polypeptide was observed in almost all serum fractions in breast cancer. In sera from prostate cancer patients, the majority of TK1 protein eluted with a peak at approximately 100 kDa, and only a minor fraction eluted in the high-MW form. These results strongly suggest that serum TK1 is present in many oligomeric forms containing both active and inactive TK1 protein. The high-MW oligomers apparently have a higher proportion of active TK1 protein compared with those with lower molecular weights. The results clearly show that there is a larger fraction of inactive TK1 protein in sera from breast and prostate cancer patients; however, even in this case, there is apparently a mixture of both active and inactive TK1 in the fractions, thus explaining the lack of co-elution between TK1 activity and protein. The varying compositions of the TK1 oligomers in sera from different patients may also explain the differences in specific activities of TK1 observed in sera from blood donors and MDS patients compared with those in sera from breast or prostate cancer patients. Specific activity indicates the active enzyme in a particular concentration of protein. To determine the specific activity of TK1 in different malignancies, both STK1 activity and TK1 sub unit of 25 kDa protein levels were measured in clinical samples. In the MDS sera, STK1 activity follows the STK1 protein profile, but there was no complete correlation between the activity and 25 kDa protein in all fractions (Additional file 1: Figure S5A). In case of healthy, there was only one peak in a high MW complex form and we could not observe any protein bands in the western blot analysis, indicating that the protein levels were very low in the fractions. These results indicate that TK1 elution profile based on TK1 activity or TK1 protein are similar and that most of the TK1 oligomers are active, resulting in high specific activity (active TK1/ mg of TK1 25 kDa protein). Whereas in breast and prostate cancer sera, the TK1 elution profile was different and the TK1 activity eluted as a major peak with high MW, while the TK1 protein eluted in multimeric forms with different MW, representing both active and inactive forms of TK1 (Additional file 1: Figure S5B and S5C). A recent study was done on comparison of TK1 molecular forms in sera from healthy dogs, dogs with leukemia and mammary tumors. The results showed that TK1 exits as high MW oligomers which are active in both healthy and leukemia sera. TK1 protein also follows the similar pattern in both cases. However, in mammary tumor sera, TK1 activity eluted as a major peak with high MW complex, while the TK1 protein eluted in multimeric forms with different MW, representing both active and inactive forms of TK1 [38]. These results are similar to that what we observed in human TK1 forms in different sera. These differences in molecular forms lead to lower specific activity as shown in Table 2. These findings are most likely due to modifications of TK1 in sera from patients with different cancer diseases. Attempts to define these presumed structural differences are ongoing and may lead to the development of an in vitro diagnostic test specific for serum TK1 in patients with different tumor diseases.

## Conclusions

Methods based on anti-TK1 antibodies and ^3^H-dThd phosphorylation for determining the specific activity of serum TK1 strongly indicate that there is a large fraction of inactive TK1, particularly in sera from patients with breast and prostate cancer. The results shown here strongly indicate that STK1 protein assays are more sensitive for the detection of early stages of breast and prostate cancer compared with STK1 activity measurements. Size-exclusion analysis of the molecular forms of STK1 demonstrated that more active forms of STK1 are found as high-molecular-weight oligomers whereas more inactive forms of STK1 protein are found as intermediate and low-molecular-weight complexes. This information is important for the future development of TK1 assays, which are more efficient for detecting early stages of common tumor diseases.

## Additional files

Additional file 1: Figure S1. Diagrammatic representation of Immunoaffinity/western blot analysis procedure. **Figure S2.** Immunoaffinity/western blot analysis of remaining serum samples from blood donors (A), MDS patients (B). **Figure S3.** Immunoaffinity/western blot results of remaining serum samples from (A) breast cancer and (B) prostate cancer patients. **Figure S4.** Size-exclusion chromatographic analysis. (A) Thymidine kinase 1 activity in serum fractions from MDS patient (Sample no. 15). (B) Western blotting analysis of MDS serum fractions using the immunoaffinity method. (C) Thymidine kinase 1 activity in serum fractions from a patient with breast cancer (Sample no. 20). (D) Western blotting analysis of serum fractions from a patient with breast cancer using the immunoaffinity method. (E) Thymidine kinase 1 activity in serum fractions from a patient with prostate cancer (Sample no. 27). (F) Western blotting analysis of serum fractions from a prostate cancer patient using the immunoaffinity method. (G) Thymidine kinase 1 activity in fractions from the serum of a blood donor (Sample 17). The numbers denote FPLC fractions. **Figure S5.** Comparison of STK1 activity and STK1 protein (A.U) in the corresponding FPLC fractions of A) MDS patient B) breast cancer and C) prostate cancer patient sera. 
Additional file 2: Table S1. Serum thymidine kinase 1 (STK1) activity, STK1 concentration, and TK1 specific activity in sera from blood donors. **Table S2.** Serum thymidine kinase 1 (STK1) activity, STK1 concentration, and TK1 specific activity in sera from Myelodysplastic syndrome (MDS) patients. **Table S3.** Serum thymidine kinase 1 (STK1) activity, STK1 concentration, and TK1 specific activity in sera from breast cancer patients. **Table S4.** Serum thymidine kinase 1 (STK1) activity, STK1 concentration, and TK1 specific activity in sera from prostate cancer patients.

## Abbreviations

MDS: Myelodysplastic syndrome
ANOVA: Analysis of variance
AUC: Area under the curve
CI: Confidence interval
CNBr: Cyanogen bromide
DNA: Deoxyribonucleic acid
dThd: Deoxythymidine
dTMP: deoxythymidine monophosphate
DTT: Dithiothreitol
FPLC: Fast protein liquid chromatography
HEPES: Hydroxyethyl-piperazineethane-sulfonic acid
MW: Molecular weight
ROC: Receiver operating characteristic
RPM: Revolution per minute
SD: Standard deviation
SDS: Sodium dodecyl sulfate
SDS-PAGE: Sodium dodecyl sulfate polyacrylamide gel electrophoresis
STK1: Serum thymidine kinase 1
TBS: Tris-buffered saline
