# Supplementary material for: Breast and prostate cancer patients differ significantly in their serum Thymidine kinase 1 (TK1) specific activities compared with those hematological malignancies and blood donors: implications of using serum TK1 as a biomarker
Source: BMC Cancer. 2015 Feb 18;15:66. doi: 10.1186/s12885-015-1073-8 (PMC4336758; doi:10.1186/s12885-015-1073-8)
Supplement: Additional file 1: Figure S1. — Diagrammatic representation of Immunoaffinity/western blot analysis procedure. Figure S2. Immunoaffinity/western blot analysis of remaining serum samples from blood donors (A), MDS patients (B). Figure S3. Immunoaffinity/western blot results of remaining serum samples from (A) breast cancer and (B) prostate cancer patients. Figure S4. Size-exclusion chromatographic analysis. (A) Thymidine kinase 1 activity in serum fractions from MDS patient (Sample no. 15). (B) Western blotting analysis of MDS serum fractions using the immunoaffinity method. (C) Thymidine kinase 1 activity in serum fractions from a patient with breast cancer (Sample no. 20). (D) Western blotting analysis of serum fractions from a patient with breast cancer using the immunoaffinity method. (E) Thymidine kinase 1 activity in serum fractions from a patient with prostate cancer (Sample no. 27). (F) Western blotting analysis of serum fractions from a prostate cancer patient using the immunoaffinity method. (G) Thymidine kinase 1 activity in fractions from the serum of a blood donor (Sample 17). The numbers denote FPLC fractions. Figure S5. Comparison of STK1 activity and STK1 protein (A.U) in the corresponding FPLC fractions of A) MDS patient B) breast cancer and C) prostate cancer patient sera. [file 12885_2015_1073_MOESM1_ESM.pptx]

## Slide 1
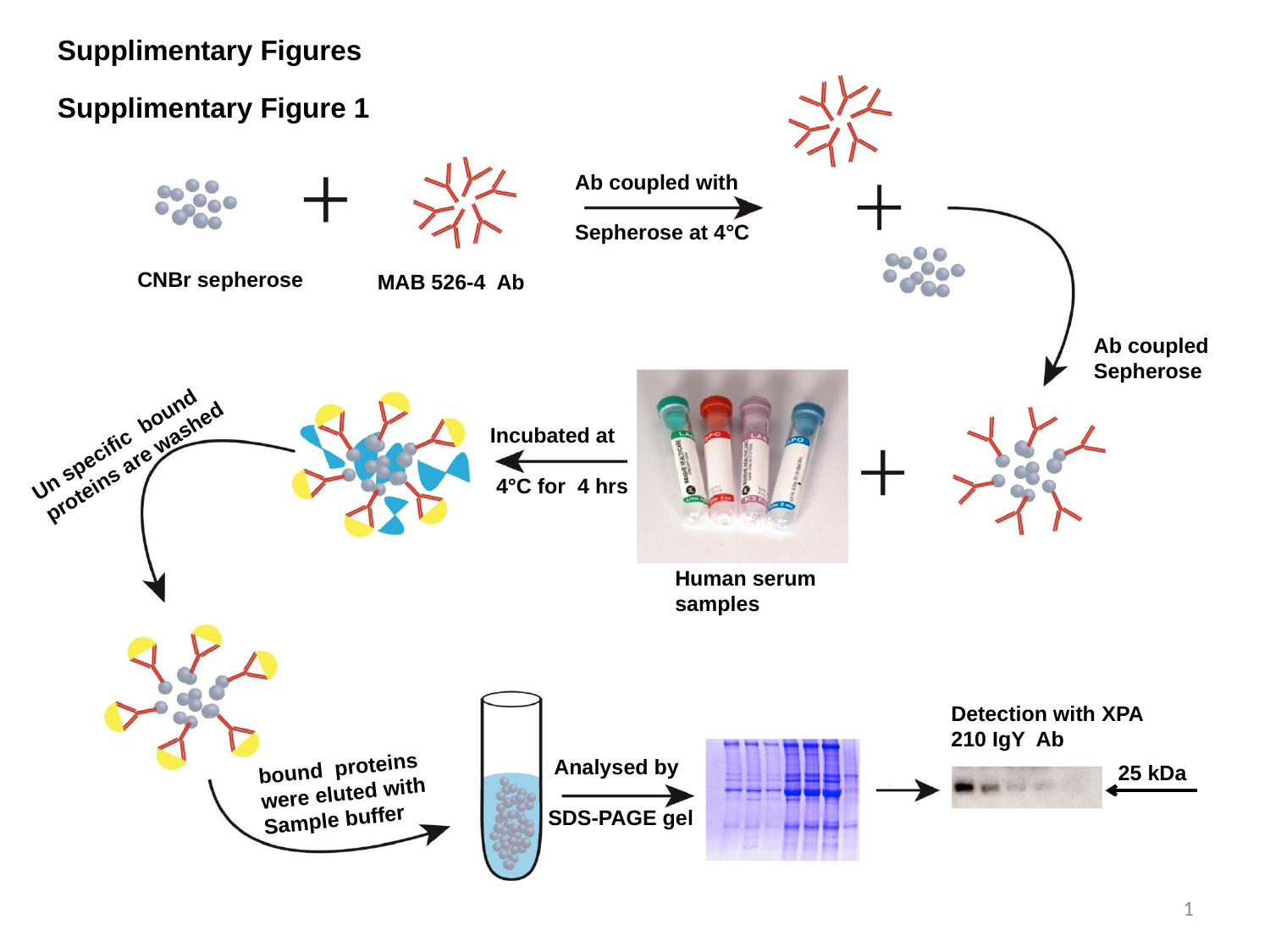

Supplimentary Figures
Supplimentary Figure 1
Ab coupled with
Sepherose at 4°C
CNBr sepherose
MAB 526-4 Ab
Ab coupled Sepherose
Un specific bound proteins are washed
Incubated at
 4°C for 4 hrs
Human serum samples
Detection with XPA 210 IgY Ab
 Analysed by
 SDS-PAGE gel
bound proteins were eluted with Sample buffer
25 kDa
1

## Slide 2
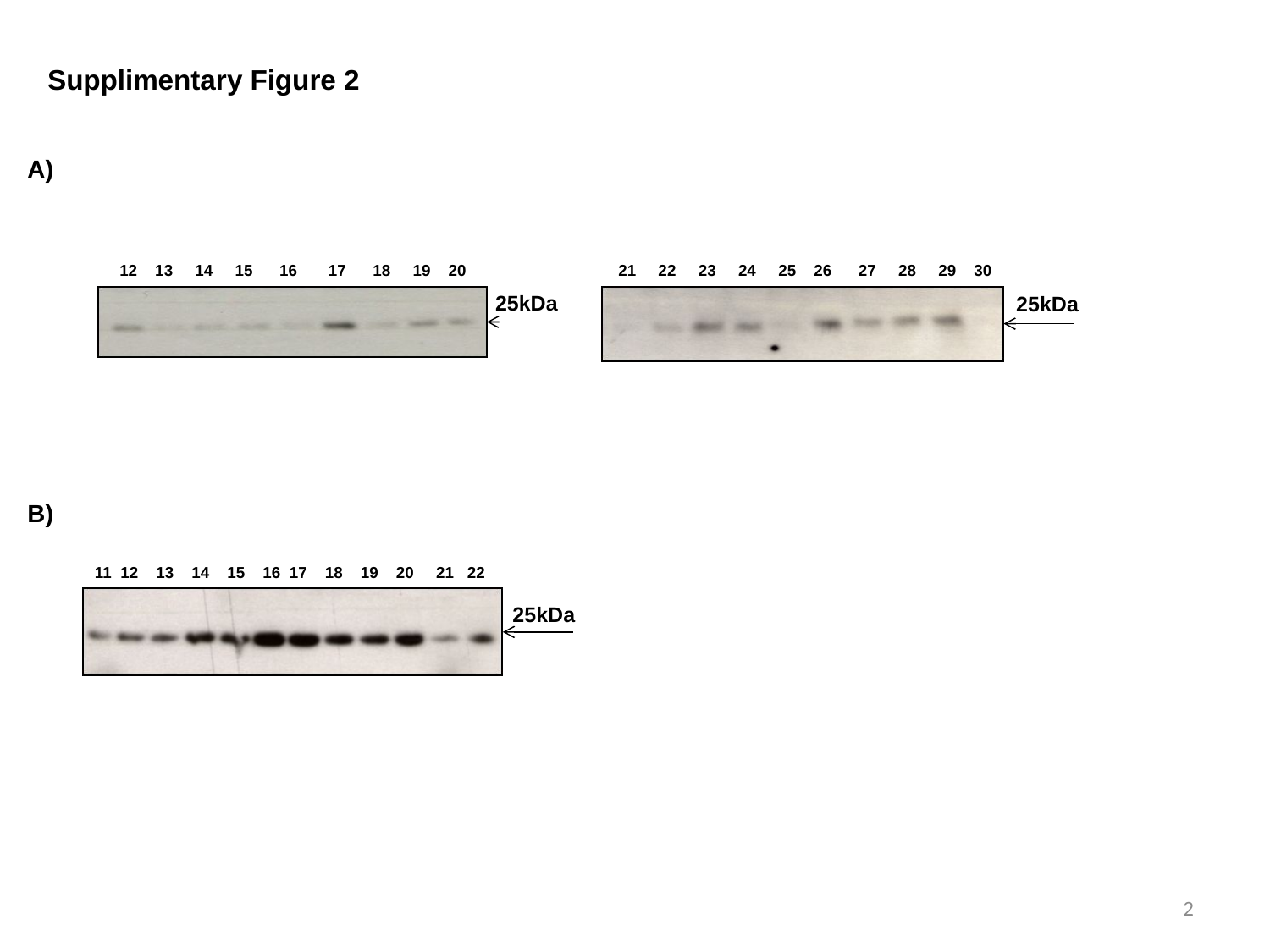

Supplimentary Figure 2
A)
 12 13 14 15 16 17 18 19 20
 21 22 23 24 25 26 27 28 29 30
25kDa
25kDa
B)
 11 12 13 14 15 16 17 18 19 20 21 22
25kDa
2

## Slide 3
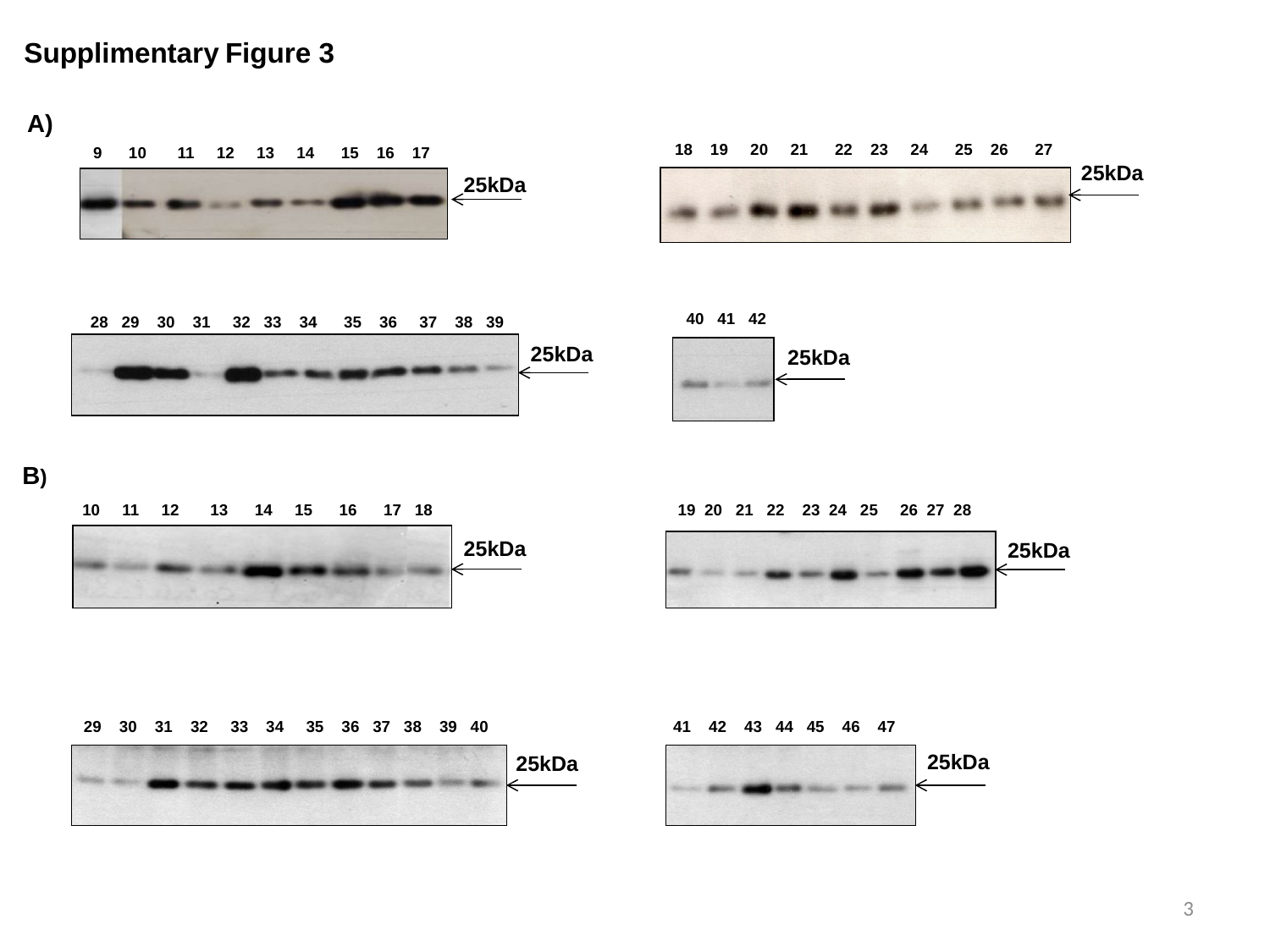

Supplimentary Figure 3
A)
18 19 20 21 22 23 24 25 26 27
9 10 11 12 13 14 15 16 17
25kDa
25kDa
 40 41 42
 28 29 30 31 32 33 34 35 36 37 38 39
25kDa
25kDa
B)
 10 11 12 13 14 15 16 17 18
 19 20 21 22 23 24 25 26 27 28
25kDa
25kDa
41 42 43 44 45 46 47
 29 30 31 32 33 34 35 36 37 38 39 40
25kDa
25kDa
3

## Slide 4
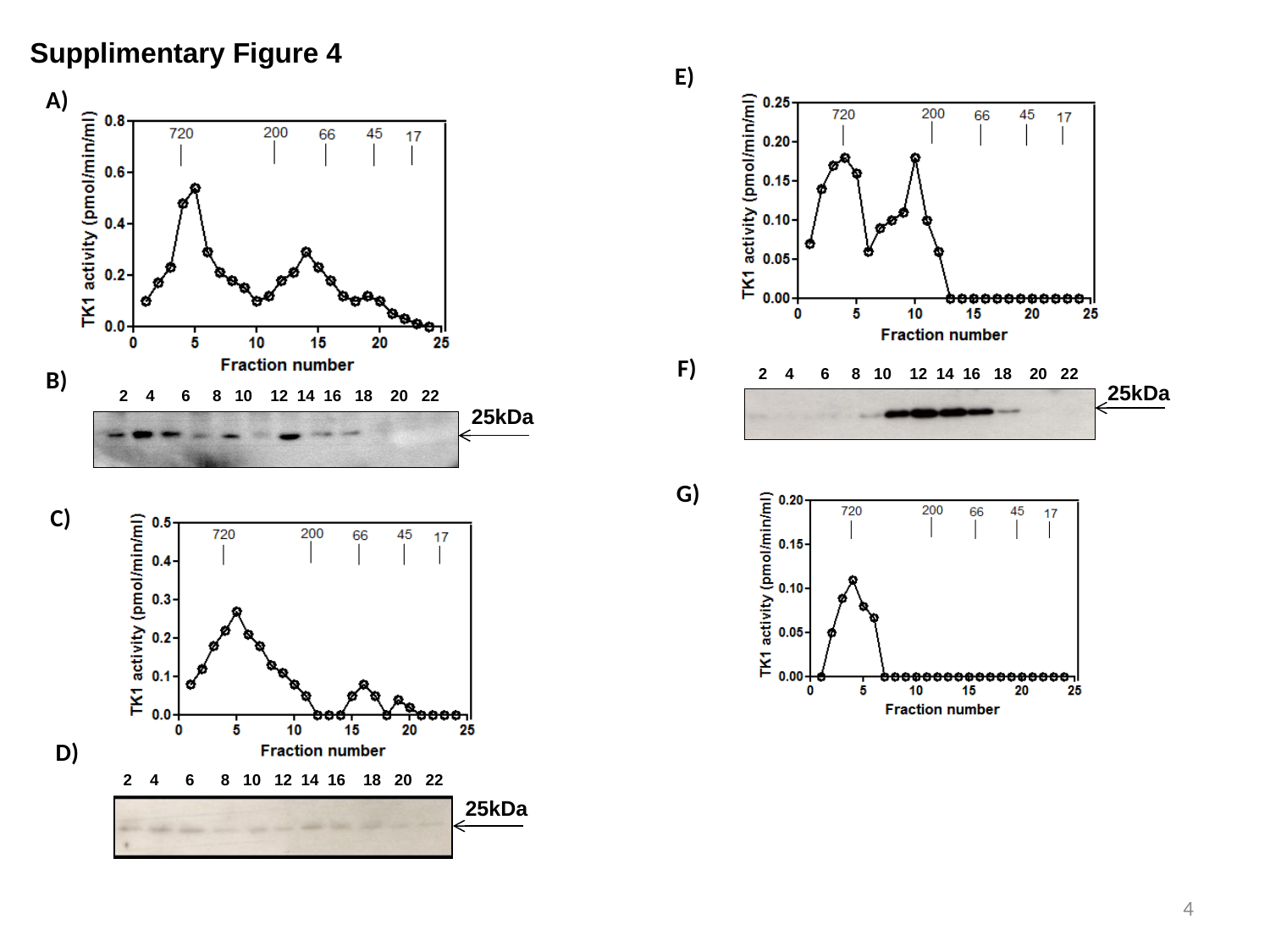

Supplimentary Figure 4
E)
A)
F)
 2 4 6 8 10 12 14 16 18 20 22
B)
25kDa
 2 4 6 8 10 12 14 16 18 20 22
25kDa
G)
C)
D)
2 4 6 8 10 12 14 16 18 20 22
25kDa
4

## Slide 5
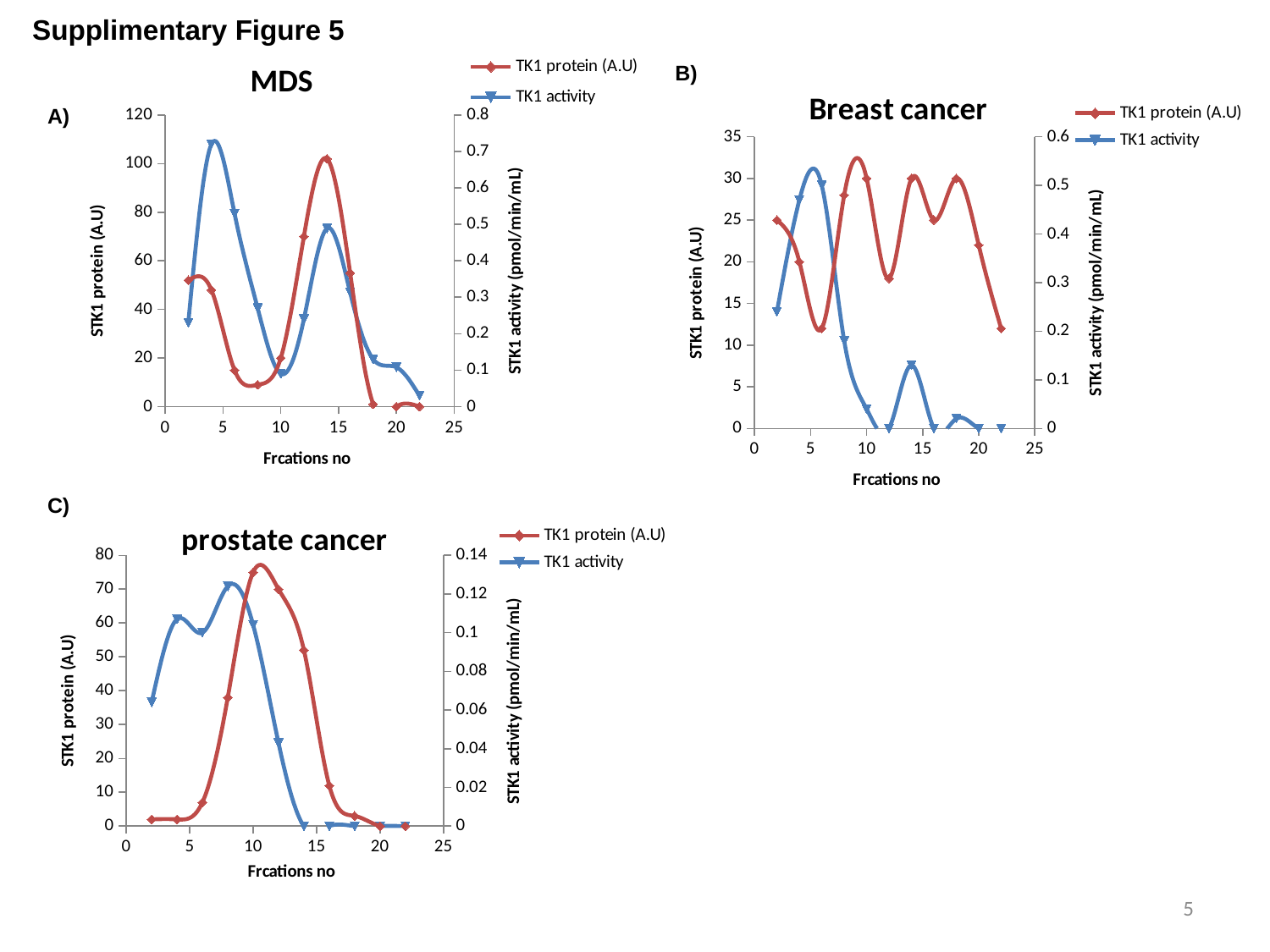

Supplimentary Figure 5
### Chart: MDS
| Category | TK1 protein (A.U) | TK1 activity |
|---|---|---|B)
### Chart: Breast cancer
| Category | TK1 protein (A.U) | TK1 activity |
|---|---|---|A)
C)
### Chart: prostate cancer
| Category | TK1 protein (A.U) | TK1 activity |
|---|---|---|5
