# Supplementary material for: Breast and prostate cancer patients differ significantly in their serum Thymidine kinase 1 (TK1) specific activities compared with those hematological malignancies and blood donors: implications of using serum TK1 as a biomarker
Source: BMC Cancer. 2015 Feb 18;15:66. doi: 10.1186/s12885-015-1073-8 (PMC4336758; doi:10.1186/s12885-015-1073-8)
Supplement: Additional file 2: Table S1. — Serum thymidine kinase 1 (STK1) activity, STK1 concentration, and TK1 specific activity in sera from blood donors. Table S2. Serum thymidine kinase 1 (STK1) activity, STK1 concentration, and TK1 specific activity in sera from Myelodysplastic syndrome (MDS) patients. Table S3. Serum thymidine kinase 1 (STK1) activity, STK1 concentration, and TK1 specific activity in sera from breast cancer patients. Table S4. Serum thymidine kinase 1 (STK1) activity, STK1 concentration, and TK1 specific activity in sera from prostate cancer patients. [file 12885_2015_1073_MOESM2_ESM.docx]

**Supplementary table 1:**  **Serum thymidine kinase 1 (STK1) activity, STK1 concentration, and TK1 specific activity in sera from blood donors.**

| **Blood donor** |  |  | **STK1 Activity** | **STK1 concentration** | **Specific activity** |
| --- | --- | --- | --- | --- | --- |
| **samples** | **Sex** | **Age** | **(pmol/min/mL)ͣ** | **(ng/mL) ᵇ** | **(nmol/min/mg)** |
|  | **(M/F)** |  | **(Mean± SD)** | **(Mean± SD)** |  |
|  |  |  |  |  |  |
|  |  |  |  |  |  |
| 1 | F | 19 | 1.64±0.05 | 11±3 | 75 |
| 2 | F | 17 | 1.06±0.08 | 6±2 | 88 |
| 3 | F | 17 | 1.31±0.03 | 3±1 | 217 |
| 4 | F | 17 | 0.92±0.04 | 3±1 | 153 |
| 5 | F | 30 | 1.12±0.05 | 3±2 | 187 |
| 6 | M | 59 | 0.89±0.04 | 2±1 | 223 |
| 7 | F | 21 | 1.23±0.07 | 10±3 | 62 |
| 8 | M | 47 | 0.73±0.04 | 3±1 | 122 |
| 9 | F | 27 | 1.19±0.08 | 4±2 | 149 |
| 10 | F | 25 | 0.53±0.05 | 2±1 | 133 |
| 11 | M | 28 | 0.84±0.1 | 2±1 | 210 |
| 12 | F | 30 | 0.85±0.05 | 5±1 | 85 |
| 13 | F | 18 | 0.97±0.04 | 2±1 | 243 |
| 14 | M | 29 | 1.58±0.08 | 3±1 | 263 |
| 15 | F | 18 | 1.59±0.09 | 3±1 | 265 |
| 16 | M | 18 | 0.69±0.01 | 2±1 | 173 |
| 17 | M | 57 | 2.46±0.08 | 11±3 | 112 |
| 18 | M | 18 | 0.72±0.06 | 2±1 | 180 |
| 19 | F | 38 | 1.51±0.06 | 5±2 | 151 |
| 20 | F | 17 | 0.73±0.08 | 4±1 | 91 |
| 21 | M | 18 | 0.49±0.03 | 2±1 | 123 |
| 22 | F | 39 | 0.57±0.03 | 4±2 | 71 |
| 23 | M | 50 | 0.61±0.04 | 10±2 | 30 |
| 24 | M | 17 | 0.45±0.03 | 6±2 | 38 |
| 25 | F | 23 | 0.98±0.07 | 4±1 | 123 |
| 26 | M | 23 | 1.21±0.08 | 12±3 | 50 |
| 27 | F | 18 | 0.81±0.02 | 7±2 | 58 |
| 28 | M | 17 | 1.45±0.09 | 10±3 | 73 |
| 29 | F | 19 | 3.23±0.11 | 13±4 | 124 |
| 30 | M | 36 | 0.52±0.02 | 2±1 | 130 |

ͣ Mean values of three observations from a single experiment. ᵇ Mean values of two observations from two independent experiments.

**Supplementary table 2:**  **Serum thymidine kinase 1 (STK1) activity, STK1 concentration, and TK1 specific activity in sera from Myelodysplastic syndrome (MDS) patients.**

| **MDS** |  |  | **STK1 Activity** | **STK1 concentration** | **Specific Activity** |
| --- | --- | --- | --- | --- | --- |
| **samples** | **Sex** | **Age** | **(pmol/min/mL)** | **(ng/mL)** | **(nmol/min/mg)** |
|  |  |  | **(Mean± SD)ͣ** | **(Mean± SD)ᵇ** |  |
|  |  |  |  |  |  |
| 1 | M | 80 | 2.84±0.12 | 14±3 | 101 |
| 2 | F | 74 | 5.44±0.32 | 27±5 | 101 |
| 3 | M | 84 | 2.32±0.24 | 17±4 | 68 |
| 4 | F | 78 | 4.42±0.56 | 12±3 | 184 |
| 5 | F | 81 | 1.10±0.09 | 8±2 | 69 |
| 6 | M | 68 | 11.3±1.42 | 22±4 | 251 |
| 7 | F | 88 | 3.52±0.47 | 18±4 | 99 |
| 8 | M | 73 | 1.21±0.08 | 3±1 | 173 |
| 9 | M | 79 | 2.76±0.19 | 28±4 | 49 |
| 10 | F | 71 | 51.2±4.56 | 30±7 | 853 |
| 11 | F | 89 | 3.06±0.15 | 7±2 | 211 |
| 12 | M | 85 | 1.85±0.07 | 11±3 | 81 |
| 13 | M | 85 | 4.24±0.21 | 12±3 | 180 |
| 14 | F | 79 | 1.45±0.06 | 24±4 | 30 |
| 15 | F | 62 | 23.7±2.23 | 23±3 | 858 |
| 16 | M | 79 | 61.8±4.56 | 36±5 | 515 |
| 17 | F | 62 | 11.8±3.11 | 30±4 | 197 |
| 18 | F | 83 | 1.71±0.21 | 22±5 | 39 |
| 19 | M | 81 | 1.51±0.05 | 20±5 | 38 |
| 20 | F | 64 | 42.2±4.31 | 29±4 | 728 |
| 21 | F | 66 | 1.79±0.09 | 8±2 | 128 |
| 22 | F | 61 | 1.95±0.11 | 12±2 | 83 |

ͣ Mean values of three observations from a single experiment. ᵇ Mean values of two observations from two independent experiments.

**Supplementary table 3:** **Serum thymidine kinase 1 (STK1) activity, STK1 concentration, and TK1 specific activity in sera from breast cancer patients.**

|  |  |  |  | **STK1 Activity** | **STK1 concentration** |  |
| --- | --- | --- | --- | --- | --- | --- |
| **Breast Cancer** | **Age** | **Staging** |  | **(pmol/min/mL)** | **(ng/mL)** | **Specific Activity** |
| **samples** |  | **(TNM)** | **Histology** | **(Mean± SD)ͣ** | **(Mean± SD)ᵇ** | **(nmol/min/mg)** |
|  |  |  |  |  |  |  |
| 1 | 83 | T1N2M1 | ID | 0.72±0.04 | 23±5 | 16 |
| 2 | 83 | T1N2M1 | ID | 0.59±0.02 | 17±3 | 17 |
| 3 | 84 | T1N2M1 | ID | 0.82±0.05 | 21±3 | 20 |
| 4 | 78 | TiSNOM2 | DCIS | 1.22±0.11 | 28±4 | 22 |
| 5 | 47 | T2NOM1 | ID | 5.95±0.21 | 37±5 | 80 |
| 6 | 43 | T2NOM1 | INVD | 3.19±0.14 | 27±3 | 59 |
| 7 | 57 | T2NOM2 | ID/L | 1.91±0.12 | 16±3 | 59 |
| 8 | 57 | T2NOM2 | ID/L | 5.29±0.24 | 16±3 | 165 |
| 9 | 64 | T2N1M1 | ID/L | 118±10 | 38±6 | 1560 |
| 10 | 57 | T2NOM2 | ID/L | 4.58±0.14 | 23±4 | 100 |
| 11 | 56 | T2N2M2 | ID | 1.62±0.09 | 24±3 | 34 |
| 12 | 66 | T3NOM1 | ID | 1.22±0.08 | 7±3 | 87 |
| 13 | 44 | T3N2M1 | ID/L | 15.5±1.2 | 29±4 | 268 |
| 14 | 63 | --- | D | 0.71±0.03 | 12±3 | 30 |
| 15 | 68 | T3NOM2 | ID | 1.84±0.13 | 38±6 | 24 |
| 16 | 47 | T2NOM1 | ID | 0,89±0,06 | 37±8 | 12 |
| 17 | 47 | T2NOM1 | ID | 0.94±0,04 | 34±5 | 14 |
| 18 | 82 | T1N1M1 | ID | 0.97±0.06 | 19±4 | 26 |
| 19 | 82 | T1N1M1 | ID | 0.86±0.05 | 16±3 | 27 |
| 20 | 46 | TiSN2M2 | DCIS | 8.46±0.56 | 28±4 | 151 |
| 21 | 83 | T4N3M1 | ID | 1.54±0.12 | 27±3 | 29 |
| 22 | 43 | T3N2M1 | DCIS | 1.12±0.11 | 20±4 | 28 |
| 23 | 46 | TisN2M2 | DCIS | 1.84±0.09 | 24±5 | 38 |
| 24 | 86 | --- | ID | 0.51±0.03 | 12±3 | 21 |
| 25 | 69 | --- | DCIS | 0.71±0.04 | 16±3 | 22 |
| 26 | 46 | TisN2M2 | DCIS | 1.03±0.11 | 16±2 | 32 |
| 27 | 46 | TiSN2M2 | DCIS | 2.54±0.14 | 19±5 | 67 |
| 28 | 43 | T1AN2M1 | DCIS | 1.51±0.08 | 5±1 | 145 |
| 29 | 64 | T2N1M2 | ID/L | 17.3±0.63 | 42±7 | 208 |
| 30 | 45 | T2N1M2 | ID/L | 2.62±0.11 | 35±5 | 38 |
| 31 | 43 | T1AN2M1 | DCIS | 1.63±0.07 | 7±2 | 116 |
| 32 | 64 | T2N1M2 | ID/L | 9.21±0.24 | 45±8 | 102 |
| 33 | 57 | T2N2M1 | IL | 1.52±0.08 | 20±4 | 37 |
|  |  |  |  |  |  |  |
| 34 | 47 | T2NOM1 | ID | 2.26±0.13 | 21±3 | 53 |
| 35 | 47 | T2NOM1 | ID | 2.31±0.19 | 28±5 | 42 |
| 36 | 47 | T2NOM1 | ID | 5.54±0.32 | 25±4 | 112 |
| 37 | 46 | TisN2M2 | DCIS | 39.5±1.65 | 23±5 | 859 |
| 38 | 57 | T2NOM2 | ID/L | 5.43±0.68 | 16±3 | 205 |
| 39 | 46 | TisN2M2 | DCIS | 2.14±0.16 | 5±2 | 202 |
| 40 | 57 | T2NOM2 | ID/L | 1.72±0,07 | 10±3 | 85 |
| 41 | 46 | TisN2M2 | DCIS | 1.94±0,11 | 4±2 | 229 |
| 42 | 46 | TisN2M2 | DCIS | 2.21±0,13 | 8±2 | 132 |

ͣ Mean values of three observations from a single experiment. ᵇ Mean values of two observations from two independent experiments.

**Supplementary table 4:**  **Serum thymidine kinase 1 (STK1) activity, STK1 concentration, and TK1 specific activity in sera from prostate cancer patients.**

|  |  |  | **STK1 Activity** | **STK1 concentration** | |
| --- | --- | --- | --- | --- | --- |
| **Prostate Cancer** | **Age** | **Gleason score** | **(pmol/min/mL)** | **(ng/mL)** | **Specific Activity** |
| **samples** |  | **(GS)** | **(Mean± SD)ͣ** | **(Mean± SD)ᵇ** | **(nmol/min/mg)** |
|  |  |  |  |  |  |
| 1 | 89 | GS6 | 1.61±0.04 | 24±4 | 34 |
| 2 | 90 | GS6 | 1.37±0.08 | 31±3 | 22 |
| 3 | 90 | GS6 | 1.12±0.09 | 27±3 | 21 |
| 4 | 71 | GS7 | 1.19±0.05 | 25±4 | 24 |
| 5 | 71 | GS7 | 1.87±0.08 | 22±3 | 43 |
| 6 | 71 | GS7 | 0.68±0.04 | 30±4 | 11 |
| 7 | 72 | GS9 | 5.91±0.12 | 21±3 | 141 |
| 8 | 90 | GS8 | 1.57±0.21 | 14±2 | 56 |
| 9 | 84 | GS6 | 0.77±0.03 | 5±2 | 77 |
| 10 | 80 | GS7 | 0.95±0.04 | 11±3 | 43 |
| 11 | 81 | GS5 | 0.85±0.02 | 9±2 | 47 |
| 12 | 83 | GS5 | 1.12±0.05 | 22±3 | 25 |
| 13 | 84 | GS5 | 0.76±0.09 | 19±4 | 20 |
| 14 | 55 | GS7 | 1.88±0.11 | 35±6 | 27 |
| 15 | 71 | GS4 | 0.69±0.04 | 28±3 | 12 |
| 16 | 84 | GS6 | 0.79±0.03 | 25±4 | 16 |
| 17 | 33 | GS6 | 0.71±0.04 | 13±3 | 27 |
| 18 | 80 | GS7 | 0.82±0.08 | 22±3 | 19 |
| 19 | 84 | GS5 | 0.64±0.05 | 7±2 | 46 |
| 20 | 56 | GS7 | 1.98±0.16 | 30±4 | 33 |
| 21 | 57 | T1NM1 | 2.71±0.12 | 10±3 | 135 |
| 22 | 88 | GS8 | 2.32±0.11 | 22±4 | 53 |
| 23 | 65 | GS7 | 0.95±0.08 | 6±3 | 79 |
| 24 | 66 | GS8 | 0.96±0.06 | 4±2 | 120 |
| 25 | 86 | GS7 | 1.59±0.07 | 11±3 | 72 |
| 26 | 75 | GS8 | 3.54±0.11 | 29±4 | 61 |
| 27 | 75 | GS8 | 6.99±0.25 | 32±5 | 109 |
| 28 | 75 | GS8 | 4.32±0.18 | 36±7 | 60 |
| 29 | 68 | T2NXM0 | 1.01±0.05 | 5±2 | 100 |
| 30 | 49 | GS9 | 1.11±0.08 | 6±2 | 92 |
| 31 | 58 | GS7 | 1.45±0.05 | 38±5 | 18 |
| 32 | 61 | -- | 1.32±0.06 | 31±4 | 21 |
| 33 | 58 | -- | 1.67±0.09 | 35±6 | 23 |
| 34 | 55 | GS7 | 4.56±0.29 | 41±4 | 55 |
|  |  |  |  |  |  |
| 35 | 67 | T2NXM0 | 1.41±0.07 | 39±6 | 21 |
| 36 | 63 | -- | 1.32±0.06 | 41±8 | 16 |
| 37 | 56 | GS7 | 3.96±0.38 | 28±6 | 70 |
| 38 | 64 | GS7 | 1.52±0.09 | 20±4 | 38 |
| 39 | 50 | GS9 | 1.14±0.09 | 12±3 | 46 |
| 40 | 55 | GS7 | 2.32±0.11 | 15±3 | 77 |
| 41 | 67 | -- | 1.02±0,09 | 3±1 | 167 |
| 42 | 60 | -- | 1.23±0,08 | 5±2 | 120 |
| 43 | 60 | GS10 | 1.15±0,11 | 3±1 | 183 |
| 44 | 62 | GS7 | 1.31±0,15 | 7±2 | 93 |
| 45 | 58 | T1N2MX | 1.12±0,07 | 3±1 | 183 |
| 46 | 91 | GS7 | 3.45±0,22 | 22±4 | 77 |
| 47 | 70 | GS6 | 1.16±0,09 | 5±2 | 110 |

ͣ Mean values of three observations from a single experiment. ᵇ Mean values of two observations from two independent experiments.
